# Effect of the Dopant Configuration on the Electronic Transport Properties of Nitrogen-Doped Carbon Nanotubes

**DOI:** 10.3390/nano12020199

**Published:** 2022-01-07

**Authors:** Kim Eklund, Antti J. Karttunen

**Affiliations:** Department of Chemistry and Materials Science, Aalto University, FI-00076 Aalto, Finland; kim.eklund@aalto.fi

**Keywords:** carbon nanotubes, nitrogen-doped carbon nanotubes, electronic transport properties, density functional theory, quantum chemical calculations

## Abstract

Nitrogen-doped carbon nanotubes (N-CNTs) show promise in several applications related to catalysis and electrochemistry. In particular, N-CNTs with a single nitrogen dopant in the unit cell have been extensively studied computationally, but the structure-property correlations between the relative positions of several nitrogen dopants and the electronic transport properties of N-CNTs have not been systematically investigated with accurate hybrid density functional methods. We use hybrid density functional theory and semiclassical Boltzmann transport theory to systematically investigate the effect of different substitutional nitrogen doping configurations on the electrical conductivity of N-CNTs. Our results indicate significant variation in the electrical conductivity and the relative energies of the different dopant configurations. The findings can be utilized in the optimization of electrical transport properties of N-CNTs.

## 1. Introduction

Since their discovery in the 1990s, carbon nanotubes (CNTs) have been extensively studied, both theoretically and experimentally, for a wide number of applications thanks to their unique electrical, mechanical, optical, and thermal properties. The properties of CNTs can be further tailored by adjusting and tuning their electronic structure with chemical doping [1,2,3,4,5]. For example, nitrogen doping alters the electrical and chemical properties of CNTs, enabling prospective uses of CNTs, especially in the field of catalysis and energy applications [1,6]. Synthesis methods of N-doped CNTs include thermal treatment, laser ablation, and chemical vapor deposition techniques [7,8].

Understanding of the structure-property correlations of N-doped CNTs (N-CNTs) is the key for improving their performance in various applications. Nitrogen content can be measured, for example with Raman spectroscopy and X-ray photoelectron spectroscopy [5,9], and the atomic-level configuration of the N-CNTs can be probed with electron-energy loss spectroscopy [10] or nitrogen core level spectrum of X-ray photoelectron spectroscopy [11]. Similar to N-doped graphene, the N atoms in N-CNTs have configurations categorized as graphitic, pyridinic, and pyrrolic [10,12]. Furthermore, both graphitic and pyridinic sites can be oxidized to pyridone sites, constituting a further type of modification based on N-doping [13]. Here, our focus is on graphitic (substitutional) doping configurations, also referred to as a quaternary configuration.

The properties of the N-doped CNTs depend on the atomic configuration of the dopants [5,10]. Pyridinic and pyrrolic dopants induce disorder in the CNTs and result in p-type semiconducting behaviour, negating the n-type effect of the substitutional sites. Recently, treatment with superacids has been reported to selectively reduce the pyridinic and pyrrolic groups while retaining the substitutional, or quaternary, N atoms [14]. Another route to further selective and atomic-level control of dopant sites could be based on the controlled doping of the carbonaceous molecular precursors used in the synthesis of chirality-controlled CNTs [15,16]. Site control of nitrogen dopants has also recently been achieved in the synthesis of phenine-nanotube molecules from pyridine and benzene building blocks [17]. Such experimental control over the nitrogen dopant sites points out the need to discover structure-property correlations related to the atom site configuration in N-CNTs. High-level control has also already been achieved in graphene, where scanning probe spectroscopy can also be used to probe conductivity around individual N dopant sites [18,19].

Electronic and transport properties of undoped CNTs have been extensively studied [20]. The electronic properties of substitutionally doped CNTs have previously been studied computationally using density functional theory (DFT), Green’s functions, [21,22,23], and tight-binding methods [24,25,26]. Other similar low-dimensional nitrogen-carbon systems such as carbon nitride and their relevant properties, such as doping energetics, have also previously been studied with DFT [27,28]. However, the structure-property correlations between the nitrogen sites and the transport properties of N-CNTs have not been systematically investigated. Here, we use hybrid density functional methods and Boltzmann transport theory to study the relation between nitrogen sites and electronic transport properties in substitutionally N-doped CNTs.

## 2. Computational Methods

Quantum chemical calculations of N-doped CNTs were carried out with the CRYSTAL17 [29] code, using hybrid PBE0 density functional method (DFT-PBE0) with 25% Hartree–Fock exchange [30,31]. Gaussian-type triple-ζ-valence + polarization (TZVP) basis sets derived from molecular Karlsruhe def2 basis sets were used for all atoms [32,33,34]. In addition to N-CNTs, we also investigated boron-doped (B-CNTs) and nitrogen–boron-codoped CNTs (BN-CNTs). To take into account the unpaired electrons introduced by N substitution, the calculations were carried out with spin-polarized formalism. We mainly focused on zig-zag-type N-doped CNTs with chirality vector (16,0). In order to compare the results with CNTs of a larger diameter, we studied two N-doped CNTs with chirality vector (22,0).

The unit cell length of the studied N-CNTs varied between 12.71 and 12.74 Å, with 192 atoms in the cell. Such supercell enables the investigation of several dopant configurations, and the dopants in the neighboring unit cells are not so close that they would complicate the interpretation of the results. We used three *k*-points in the irreducible Brillouin zone (IBZ) for sampling the reciprocal space. Five *k*-points were used for the evaluation of the Fermi energy. Fermi smearing of 0.001 a.u. (315 K) was applied for all systems. Crystalline orbitals of the N-CNTs were investigated as Bloch functions. For the evaluation of the Coulomb and exchange integrals (TOLINTEG), tight tolerance factors of 8, 8, 8, 8, and 16 were used. In the geometry optimizations, both the atomic positions and the lattice constant *a* were fully optimized. Default optimization convergence thresholds and DFT integration grids of CRYSTAL17 were applied. Due to the large size and low symmetry of the unit cells, we did not carry out any harmonic frequency calculations. Optimized geometries of the studied systems are given as Supplementary Materials and are available in the NOMAD Repository [35].

Electron transport properties can readily be accessed by solving the Boltzmann equation from a DFT wavefunction using various code packages such as BoltzTraP [36]. Here, the calculation of electronic transport properties was carried out using semiclassical Boltzmann transport theory and analytical evaluation of band velocities in the constant relaxation time approximation as implemented in the CRYSTAL code [37]. Our focus is on the relative electronic conductivities of N-CNTs with different dopant configurations. Therefore, we used the same constant electronic relaxation time (τ) of 1 femtosecond for all studied systems and carried out the calculations at the room temperature of 300 K. Detailed investigation of the absolute electrical conductivities would require ab initio calculation of τ, which we do not pursue here. Such computations would require a explicit solution of electron–phonon scattering, which is computationally extremely expensive to pursue. Thus, the modeling methodology assumes the electron–phonon properties of each tube reported to be equal. Chemical potential and transport distribution function were evaluated from −9 to 0 eV with 0.10 eV steps. For transport property calculations, 33 *k*-points in the IBZ were used (65 for the evaluation of the Fermi energy).

## 3. Results and Discussion

### 3.1. Overview of the Studied N-CNT Systems

**Figure 1:**
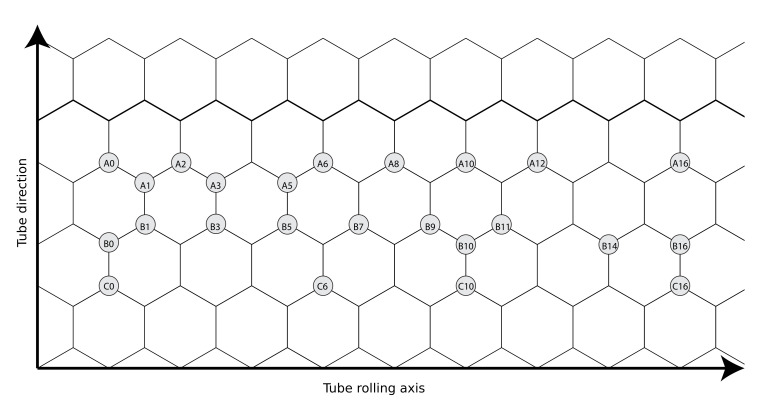
Naming scheme for the studied N-CNTs with two nitrogen-dopant atoms depicted on a surface that represents half of the supercell used in the calculations. The first N atom is always located at the position A0, and the other labels refer to the position of the second N atom. One zigzag chain is illustrated in bold.

We are interested in the doping behavior of semiconducting CNTs and use a semiconducting (16,0) zigzag CNT as the starting point. Armchair CNTs with chirality vector (n,n) are known to be metallic [38] and chiral tubes would lead in unit cells that are not computationally feasible. The naming scheme used for the investigated N-doped (16,0) CNT structures is presented in Figure 1. The illustrated structure corresponds with the unit cell size in the tube direction and half of the calculation unit cell size in the tube rolling direction. The first N atom is always located at the position A0. The second atom is located at a position along one of the zigzag-type carbon chains A, B, and C. The running integer label determines the location along the zigzag chain.

A summary of the investigated systems, along with relative energies, relative electrical conductivities at the Fermi level, and N–N distances are presented in Table 1. For the sake of comparison, CNTs with single N-dopant and single B-dopant, as well as B–N-codoped reference systems are included.

### 3.2. Relative Energies

The relative energies of the studied N-CNT systems with two dopant atoms show that the lowest-energy position for the second dopant atom is the position B0, right across the first dopant position A0 in the same six-membered ring. This configuration of two substitutional nitrogen dopants has also been identified as the lowest-energy configuration in previous studies [22]. The dopant configuration, where the second dopant atom is located at the position A1, the neighboring site to the first dopant atom, is the energetically least-favorable of all the studied configurations. The relative energetic unfavorability of such a configuration has also been discussed previously, and has been attributed to the N–N bond in principle being weaker than a C–N or a C–C bond [22]. Except for the dopant position B0, the general trend is that the dopant configurations where the second dopant atom is located further away from the first dopant are energetically more favorable. However, some fluctuation in the relative energies still occurs. For example, dopant position A6 is higher in energy (ΔE = 41 kJ/mol) compared to the more distant position A8 (ΔE = 36 kJ/mol). Another interesting observation arises when comparing positions C0 and B3, which are located about 4.2 Å from position A0, but in different directions. Site C0 (ΔE = 41 kJ/mol) in the tube direction is energetically more favorable than B3 (ΔE = 49 kJ/mol), which is shifted both in the direction of the tube and the tube rolling axis. The situation is similar for positions B1 (ΔE = 58 kJ/mol) and A2 (ΔE = 64 kJ/mol). Both are located 2.4 Å from the position A0, but the energetically less-favorable position A2 is shifted only along the tube rolling axis.

### 3.3. Electronic Transport Properties

**Figure 2:**
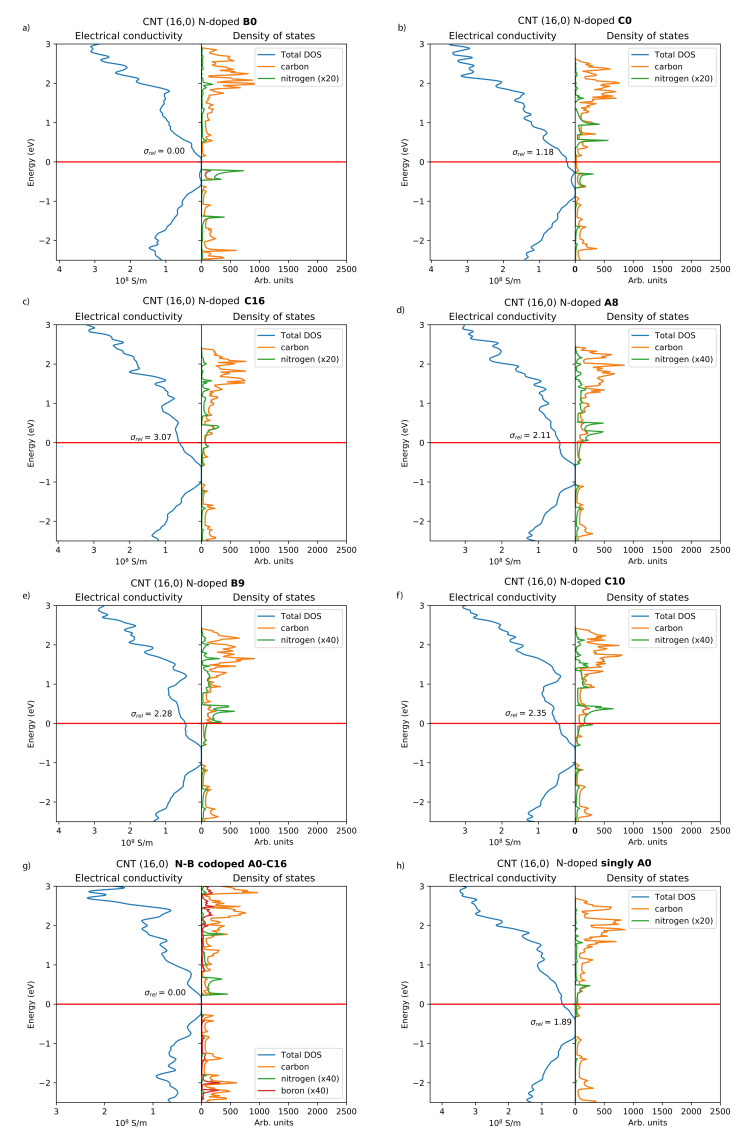
Electrical conductivities and densities of states of the studied N-CNT systems (**a**–**f**), B–N-codoped system (**g**), and single N-dopant system (**h**). Constant relaxation time τ = 1 fs was used for all systems, and only the relative magnitudes of the electrical conductivities between N-CNTs should be compared. For naming, see Figure 1.

The densities of states and electrical conductivity plots of selected N-CNT structures are presented in Figure 2. The lowest-energy configuration B0 (Figure 2a) shows negligible electrical conductivity and DOS at the Fermi level when compared to configurations where the second N atom is located further away. In this configuration, the nitrogen states are located below the Fermi level and the Fermi level essentially lies in a band gap. In addition, the energetically least-favorable position A1 shows negligible conductivity. Overall, if a second dopant is introduced in the same six-membered ring as the position A0, the electrical conductivity actually decreases in comparison to reference system containing only one dopant atom (Figure 2h). However, for the configuration B1, the electrical conductivity is already much higher compared to B0 or A1.

Besides the configuration B0, the DOS and electrical conductivity plots of the N-CNTs with two dopant atoms (Figure 2b–f) show n-type conductivity, as expected [5,14]. Generally, the electrical conductivities increase when the separation between the two dopants increases. For the configuration C0 (Figure 2b), there are no nitrogen states at the Fermi level and the relative electrical conductivity is 1.18. For the most distant C-type position C16 (Figure 2c), there are nitrogen states at the Fermi level and the configuration shows the highest relative electrical conductivity of 3.07. The configurations A8, B9, and C10 illustrated in Figure 2d–f show intermediate electrical conductivities and nitrogen states at the Fermi level.

The density of states and electrical conductivity plot of a B–N-codoped CNT is included for comparison in Figure 2g. Both this and an entirely undoped system are nonconducting, the band gap being slightly smaller in the B–N-codoped CNT. This was also observed in a previous study for B–N-codoped (13,0) CNT systems [23]. Conductivity in B–N diametrically opposite codoped (17,0) CNT systems was also studied by Khalfoun et al. [24], and our results reproduce the reported decrease in the conductivity at around 0.5 eV from the Fermi level. Our conclusions based on the investigation of (16,0) CNTs are further supported by the two investigated (22,0) N-CNT systems, with electrical conductivity 1.2 times larger for a configuration having the second N atom situated at a distance of $d_{tubular}$ = 17.12 Å versus a shorter distance of $d_{tubular}$ = 12.93 Å. Both locations are along the same carbon “chain”, comparable to the “A” sites of the CNT (16,0) system.

**Figure 3:**
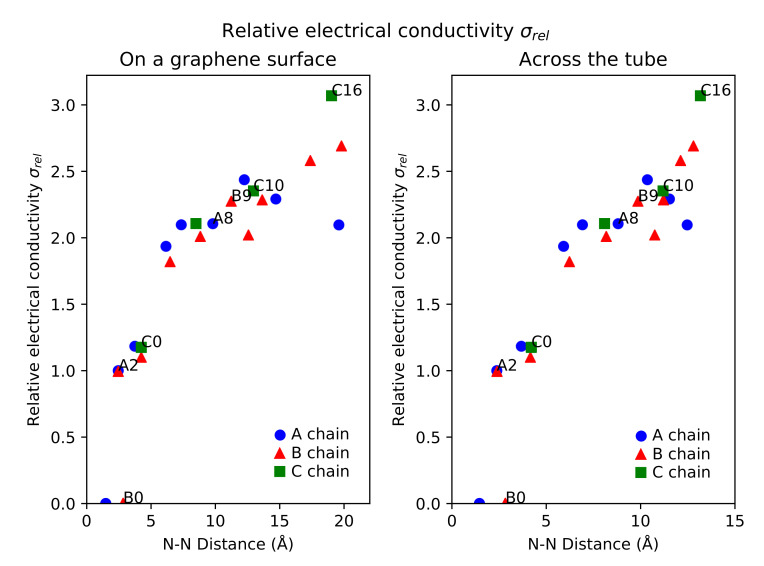
Relative electrical conductivity $\sigma_{rel}$ (S/m) of N-CNTs with respect to N–N distance between the two dopants. The plot on left is for N–N distances on an idealized graphene surface ($d_{surface}$) and the plot on the right for N–N distances across the tube ($d_{tubular}$), measured directly for the studied systems. See Figure 1 for the naming scheme. A-type positions are in blue circles, B-type positions are in red triangles, and C-type positions are in green squares.

Overall, the dopant positions along the A and B chains up to A5 and B5 have smaller electrical conductivities than the singly-doped N-CNT. The conductivities start to increase in relation to the singly-doped N-CNT when the N atoms are located about 7 Å from each other on the idealized graphene surface model. There are, however, more exceptions to the general trend, such as the site B10, where the conductivity is slightly smaller than at the surrounding sites for the second N dopant. The relationship between the N–N distances and relative electrical conductivities is illustrated in Figure 3, with both the tubular ($d_{tubular}$) and surface ($d_{surface}$) distances of Table 1 included. The plots show how, at the larger N–N distances, the relative electrical conductivities behave differently for the zig-zag chains A, B, and C. For A, $\sigma_{rel}$ for increases with the distance, but then peaks at about 12 Å and starts to decrease. For B and C, there is no such peak, but the most distant C position C16 shows larger $\sigma_{rel}$, even though the N–N distance is similar to the most distant B positions.

### 3.4. Electronic Band Structures and Crystalline Orbitals

**Figure 4:**
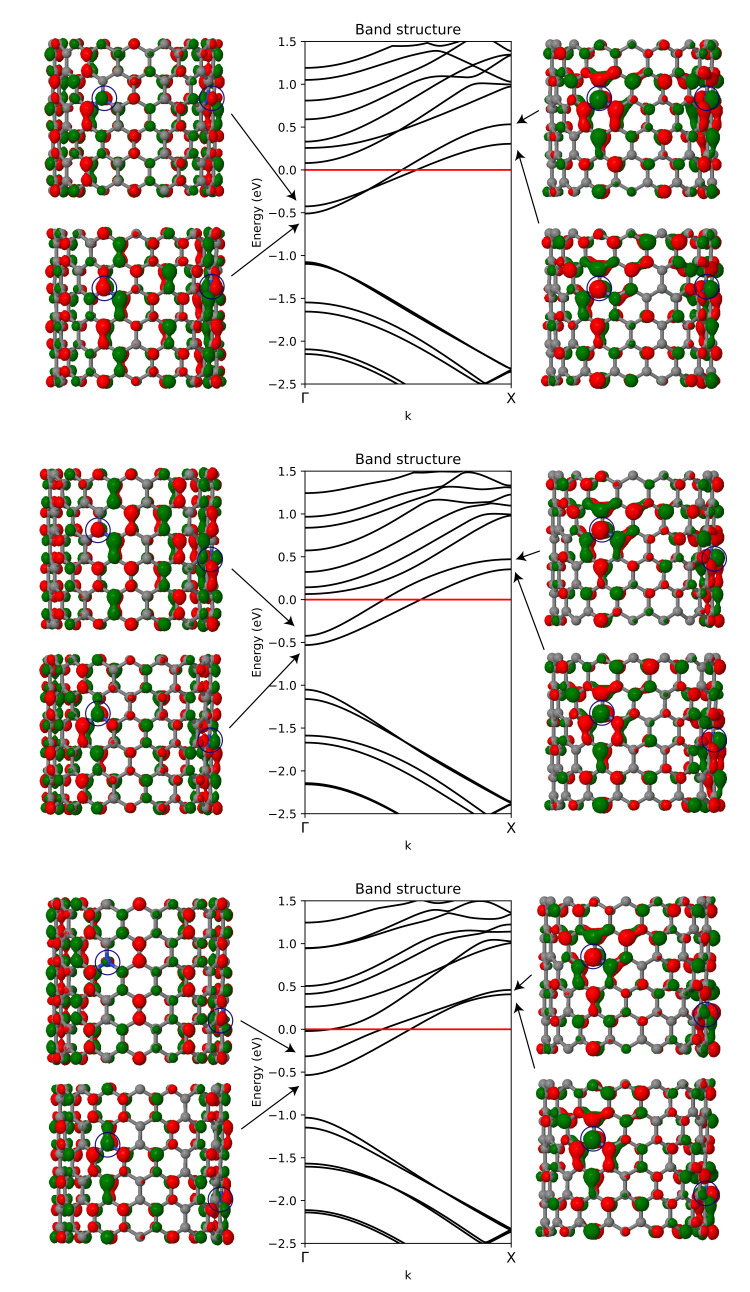
Electronic band structures of N-CNT systems A8 ($\sigma_{rel}$ = 2.11), B9 ($\sigma_{rel}$ = 2.28), and C10 ($\sigma_{rel}$ = 2.35). The location of the two nitrogen dopants is illustrated with blue circles. The crystalline orbitals associated with two bands crossing the Fermi level are shown at Γ and X points, the latter point corresponding to the boundary of the first Brillouin zone at π/2. Isodensity value of 0.02 a.u. was used to plot the crystalline orbitals.

In order to understand the effect of the substitutional nitrogen doping on the electronic band structure and crystalline orbitals of the N-CNTs, three systems were chosen for further study. The band structures and two selected crystalline orbitals of configurations A8, B9, and C10 are presented in Figure 4.

A general trend shared by all three band structures discussed here is that the energy of the bands crossing the Fermi level increases when moving from Γ to X along the *k*-path. The increasing energy of the two bands crossing the Fermi level can be explained with the crystalline orbitals plotted at Γ and X points. Focusing on the crystalline orbitals in the vicinity of the nitrogen dopant on the left, the crystalline orbitals at the X point show a larger antibonding contribution compared to Γ.

The relative electrical conductivity of the systems plotted in Figure 4 increases as A8 < B9 < C10. When moving from from A8 to B9 and to C10, the separation of the two bands crossing the Fermi level increases at the Γ point. With increased separation, the upper band is shifted closer towards the Fermi level. In C10, there is also an additional band crossing the Fermi level at the Γ point. Increase of density of states close to the Fermi level at the Γ point contributes to increased conductivity. In structure B9, there are also two bands in close proximity to the Fermi level at Γ. In configuration A8, the bands crossing the Fermi level change their relative ordering when moving, and exhibit a switching of the relative energies of the bands crossing the Fermi level when moving from Γ to X. In contrast, in B9 and C10, the ordering of the bands stays the same from Γ to X.

## 4. Conclusions

We have studied the electronic transport properties of substitutionally nitrogen-doped (16,0) carbon nanotubes using hybrid DFT methods and Boltzmann transport theory. By systematically exploring the effect of the relative positions of the nitrogen dopants, we have expanded upon the previous tight-binding-level work on the long-range effects and dopant ordering of N-CNTs [25,26]. Our results corroborate the previous computational studies pointing towards a clear effect of the dopant configuration on the electrical properties of substitutionally doped CNTs [22,23]. While experimental corroboration on CNTs is not known to us, similar observations have been made on a N-doped graphene surface looking at the increased bias voltage obtained with scanning tunneling spectroscopy when moving away from the position directly above the N dopant [18,19]. In general, the electrical conductivity of an N-CNT system with two dopant atoms increases with the separation of the N atoms, the electrical conductivity surpassing that of a singly N-doped CNT when the separation of the dopant atoms is more than ca. 7 Å. However, the N–N distance alone does not determine the electrical conductivity, as there are further exceptions arising from the relative position of the dopants along the tube axis. Generally, the electrical conductivity can vary hundreds of percent depending on the relative positions of the nitrogen dopants, motivating further experimental efforts at controlling and characterizing substitutional N-doping of CNTs at the atomic level. Recent advances in the controlled synthesis of both substitutional N-CNTs [14] and single-chirality [15] CNTs could serve as the groundwork for such experimental validation.

## Figures and Tables

**Table 1:** Relative energy (ΔE), relative electrical conductivity at Fermi level ($\sigma_{rel}$), N–N distance across the tube ($d_{tubular}$), and N–N distance on an idealized graphene surface ($d_{surface}$) for the studied systems. Naming scheme is explained in Figure 1. In addition to the N-CNTs with two nitrogen dopants, CNTs with a single N-dopant and a single B-dopant, as well as a B–N-codoped reference system are included in the bottom of the table.

N Site	ΔE	$\sigma_{\mathrm{rel}}$	$d_{\mathrm{tubular}}$	$d_{\mathrm{surface}}$
	(kJ/mol)		(Å)	(Å)
A1	139	0.00	1.46	1.41
A2	64	1.00	2.38	2.45
A3	50	1.18	3.68	3.74
A5	36	1.94	5.92	6.16
A6	41	2.10	6.92	7.35
A8	36	2.11	8.81	9.80
A10	37	2.44	10.36	12.25
A12	35	2.29	11.53	14.70
A16	34	2.10	12.47	19.60
B0	0	0.00	2.83	2.83
B1	58	0.99	2.39	2.45
B3	49	1.10	4.16	4.24
B5	40	1.82	6.23	6.48
B7	39	2.01	8.18	8.83
B9	36	2.28	9.86	11.23
B10	33	2.02	10.75	12.57
B11	34	2.28	11.21	13.64
B14	35	2.58	12.12	17.38
B16	33	2.69	12.80	19.80
C0	41	1.18	4.21	4.24
C6	39	2.11	8.09	8.49
C10	36	2.35	11.19	12.96
C16	34	3.07	13.17	19.03
single N at A0	-	1.89	-	-
single B at A0	-	1.47	-	-
B at A0, C10	-	2.29	11.60	12.96
B at A0, C16	-	2.94	13.74	19.03
N at A0, B at C16	-	0.00	13.42	19.03

## Data Availability

Optimized geometries of the studied systems are available in the NOMAD Repository [35].

## Supplementary Materials

The following are available online at https://www.mdpi.com/article/10.3390/nano12020199/s1, Optimized geometries of the studied systems.
